# Relationship between Serum 25-Hydroxyvitamin D and Lower Extremity Arterial Disease in Type 2 Diabetes Mellitus Patients and the Analysis of the Intervention of Vitamin D

**DOI:** 10.1155/2015/815949

**Published:** 2015-04-01

**Authors:** Wan Zhou, Shan-Dong Ye

**Affiliations:** Department of Endocrinology, Anhui Provincial Hospital Affiliated to Anhui Medical University, Hefei, Anhui 230001, China

## Abstract

The aim of this study was to explore the relationship between serum 25-hydroxyvitamin D [25(OH)D] concentrations and lower extremity arterial disease (LEAD) in type 2 diabetes mellitus (T2DM) patients and to investigate the intervention effect of vitamin D. 145 subjects were assigned to a control group (Group NC), T2DM group (Group DM1), and T2DM complicated with LEAD group (Group DM2); then Group DM2 were randomly divided into Group DM3 who received oral hypoglycemic agents and Group DM4 who received oral hypoglycemic drugs and vitamin D3 therapy. Compared to Group NC, 25(OH)D was significantly lower in Group DM2 and marginally lower in Group DM1. In contrast to baseline and Group DM3, 25(OH)D rose while low density lipoprotein (LDL), retinol binding protein 4 (RBP4), and HbA1c significantly lowered in Group DM4. Statistical analysis revealed that 25(OH)D had a negative correlation with RBP4, duration, HbA1c, homeostasis model assessment for insulin resistance (HOMA-IR), and fasting plasma glucose (FPG). LDL, systolic blood pressure (SBP), FPG, and smoking were risk factors of LEAD while high density lipoprotein (HDL) and 25(OH)D were protective ones. Therefore, we deduced that low level of 25(OH)D is significantly associated with the occurrence of T2DM complicated with LEAD.

## 1. Introduction

Lower extremity arterial disease (LEAD) is a common peripheral arterial disease, which seriously affects the patient's functional capacity and quality of life [1]. It is one of the factors that contribute to the progressive and the critical courses of foot ulceration and amputation in type 2 diabetes mellitus (T2DM) patients [2], and it is frequently associated with coronary, cerebral, and renal artery diseases [3]. Vitamin D is a secosteroid, which is obtained from exposure to sunlight and through dietary sources including food and supplements. It is hydroxylated in the liver to 25-hydroxyvitamin D [25(OH)D] and further hydroxylated in the kidney to form 1,25-dihydroxyvatamin D [1,25(OH)2D, calcitriol]. Although 1,25(OH)2D is considered to be the active form of vitamin D, its level in the serum does not correlate with overall vitamin D status, whereas the 25(OH)D level is a more clinically relevant marker [4]. In addition to the traditional involvement of vitamin D in bone metabolism, several lines of evidence suggest a role for vitamin D in glucose levels, insulin resistance (IR), and prevalence of T2DM [5–8], and there are more and more studies that have found that it participated in systemic inflammation, immune, and lipid metabolism to reduce the risk of cardiovascular diseases [9], but few investigations suggest the association between vitamin D and T2DM complicated with LEAD. Hence, demonstrating a relationship between 25(OH)D and T2DM complicated with LEAD is necessary. Retinol binding protein 4 (RBP4) is a new adipokine identified by Yang et al. [10] using gene chip technology, which involves the occurrence of IR, T2DM, and macrovascular complications. Several researches [11] have shown that vitamin D is correlated with adiponectin, leptin, and other adipokines, but there are few studies analyzing a relationship between 25(OH)D and RBP4 in T2DM patients complicated with LEAD. Therefore, we realize the importance of further investigation in this area. In this effort, we conducted a clinic-based case-control study to explore the relationship among 25(OH)D, RBP4, and T2DM complicated with LEAD and analyze the intervention effect of vitamin D for LEAD.

## 2. Materials and Methods

### 2.1. Subjects

107 patients (63 males and 44 females) who were recruited from outpatient department were in line with the T2DM diagnosis standard delivered by World Health Organization (WHO) in October 1999. They were hospitalized from October 2012 to January 2013 in the Department of Endocrinology in Anhui Provincial Hospital. This study was conducted in accordance with the tenets of the World Medical Association's Declaration of Helsinki and had been approved by the China Ethics Committee. Written informed consent was obtained from all participants.

The subjects who met the following criteria were excluded: (1) individuals who had acute complications of T2DM, retinopathy, nephropathy, and other chronic complications, cancer, vitamin A deficiency, liver and kidney dysfunction, osteoporosis, and other history of bone metabolic disorders; (2) those taking drugs including vitamins, calcium, lipid lowering drugs, estrogen, and other drugs affecting bone metabolism. All the patients were assigned into 2 groups: the one that is without complications (Group DM1: 25 males, 20 females) and the other that is complicated with LEAD (Group DM2: 38 males, 24 females); then subjects in Group DM2 were further divided into Group DM3 (16 males, 15 females) and Group DM4 (22 males, 9 females). Patients in these 2 groups were both treated with hypoglycemic drugs, and additionally patients of Group DM4 were orally administered vitamin D3 1000 IU daily. In this period 2 subjects in Group DM3 were out of touch and 4 subjects in Group DM4 could not insist on the intervention therapy and quitted. The relevant characteristics were tested and ABI was reexamined in Groups DM3 and DM4 after 12 weeks of therapy. 38 age-sex-matched healthy people with no hypertension or impaired glucose tolerance were chosen at the same period as a control group (Group NC) (20 males and 18 females).

### 2.2. ABI Measurement

The Doppler instrument (Bidop ES-100-v3 produced in Japan) was adapted. All the ABI measurements were performed with the subjects in the supine position, a blood pressure cuff was placed on patients' upper arms, and it was inflated until no brachial pulse was detected by the Doppler device. The cuff was then slowly deflated until the Doppler-detected pulse returned to measure brachial systolic blood pressure (BSBP). This maneuver was repeated on the leg, with the cuff being wrapped around the distal calf and the Doppler device being placed over the dorsalis pedis or the posterior tibial artery to measure ankle systolic blood pressure (ASBP) [12]. Ankle brachial index (ABI) = ASBP/BSBP and ABI < 0.9 indicated the presence of LEAD [13].

### 2.3. Assays

All the subjects took balanced diets for 3 days and then fasted overnight for 12 hours. Clinical characteristics including sex, age, diabetes duration, height, weight, hip circumference, blood pressure, and history of smoking were collected. Body mass index (BMI) and waist-hip ratio (WHR) were calculated as weight divided by height squared (kg/m^2^) and waist circumference divided by hip circumference, respectively. Systolic blood pressure (SBP) and diastolic blood pressure (DBP) were measured using a digital automatic blood pressure monitor. Patients' blood samples were kept frozen at −80°C until analysis. Fasting plasma glucose (FPG), total cholesterol (TC), triglycerides (TG), low density lipoprotein (LDL), and high density lipoprotein (HDL) were detected by Hitachi 7600 2020 automatic biochemical analyzer. HbA1C was conducted by high-pressure liquid chromatography (Bio-Rad variant turbo II analyzer). Fasting insulin (FINS) was tested by radioimmunoassay (Linco Research, St. Charles, USA). The homeostasis model assessment for insulin resistance (HOMA-IR) was calculated according to the formula HOMA-IR = FINS (mU/L) × FPG (mmol/L)/22.5. Serum 25(OH)D concentrations were detected via radioimmunoassay (Diasorin Stillwater, MN, USA). Sufficiency was indicated when 25(OH)D ≥ 30 ng/mL, insufficiency when 20 ng/mL ≤ 25(OH)D < 30 ng/mL, and deficiency when 25(OH)D < 20 ng/mL [14]. The level of RBP4 was measured by enzyme-linked immunoassay (AssayPro, MO, USA).

### 2.4. Statistical Method

All data were calculated by SPSS17.0 statistical software (SPSS Inc., Chicago, IL, USA). Continuous variables are presented as mean ± standard deviation ($\overset{-}{x}\pm s$), or median and interquartile range (25th and 75th percentile) in cases of skewed distributions, while categorical variables are presented as percentages. These groups were compared using two-sample *t*-test, Mann Whitney *U* test, or chi-square test. Paired sample *t*-test was used to compare the differences before and after treatment. Pearson's correlation coefficient or Spearman's rank correlation coefficient was used to assess the relationship between 25(OH)D and other markers. Multivariable logistic regression analysis was used to calculate the ORs and 95% confidence intervals for LEAD; statistical significance was accepted at *P* < 0.05.

## 3. Results

### 3.1. Clinical and Biochemical Characteristics

The indexes of the subjects were listed in Table 1. There were no differences of sex between the 3 groups (*P* > 0.05). Participants in Group DM2 had a longer duration than in Group DM1; the differences were statistically significant (*P* < 0.05). Serum 25(OH)D concentrations were 24.77 ± 5.9 ng/mL, 17.32 ± 7.42 ng/mL, and 12.63 ± 7.83 ng/mL in Groups NC, DM1, and DM2, respectively; compared with Group NC, 25(OH)D decreased in Groups DM1 and DM2 and the decrease in Group DM2 was more significant (*P* < 0.05). RBP4 level was lower in T2DM patients with LEAD compared to the patients without LEAD and healthy people (*P* < 0.05). Raised FPG, FINS, HbA1C, TC, TG, LDL, HOMA-IR, SBP, and WHR and decreased HDL in Groups DM1 and DM2 were exhibited as compared with Group NC (*P* < 0.05); age, FPG, LDL, SBP, HbA1C, and the rate of smoking increased and HDL decreased in Group DM2 compared with the other 2 groups (*P* < 0.05).

### 3.2. Cases with Different Levels of 25(OH)D among Three Groups

As depicted in Table 2, approximately 18.4% of subjects were classified as 25(OH)D sufficient and 65.8% were classified as 25(OH)D insufficient; only 15.8% of subjects were classified as 25(OH)D deficient in Group NC. In Group DM1, only 8.9% of subjects had normal 25(OH)D level and 26.7% had insufficiency 25(OH)D level while 64.4% were 25(OH)D deficient. The percentages of 25(OH)D deficiency, insufficiency, and sufficiency were 77.4%, 14.5%, and 8.1%, respectively, in Group DM2. The percentages of sufficient 25(OH)D level in Groups DM1 and DM2 were both lower than the percentage in Group NC, and the difference was statistically significant (*P* < 0.05).

### 3.3. The Levels of Clinical Characteristics before and after the Treatment in Diabetic Groups Complicated with LEAD

There was no significant difference between Groups DM3 and DM4 at baseline. After 12 weeks of vitamin D supplementation in Group DM4, 25(OH)D level increased and LDL, RBP4, and HbA1C decreased significantly when compared with baseline (*P* < 0.05), while, following 12-week basic therapy of hypoglycemic drugs in Group DM3, only RBP4 and HbA1C decreased (*P* < 0.05). Supplementation with vitamin D for 12 weeks in diabetics with LEAD significantly raised 25(OH)D level and lowered LDL, RBP4, and HbA1c relative to baseline and unsupplemented diabetics (*P* < 0.05). Reexamination of ABI by Doppler ultrasonography for subjects in Group DM3 and Group DM4 after 12 weeks showed that there were, respectively, 5 cases in Group DM3 and 10 cases in Group DM4 whose values increased to ≥0.9, and the difference was not statistically significant (*P* > 0.05) (Table 3).

### 3.4. Correlations between 25(OH)D and Other Indexes

As depicted in Table 4 the correlation analysis results suggested that the level of 25(OH)D was inversely associated with duration (*r* = −0.663, *P* < 0.05), HbA1c (*r* = −0.482, *P* < 0.05), RBP4 (*r* = −0.538, *P* < 0.05), FPG (*r* = −0.229), and HOMA-IR (*r* = −0.267, *P* < 0.05), while there was no significant correlation between 25(OH)D and the remaining indicators (*P* > 0.05). 25(OH)D had a positive correlation trend with HDL, but the difference was not statistically significant (*P* > 0.05).

### 3.5. Independent Factors for the Presence of LEAD

As shown in Table 5, the presence of LEAD in T2DM patients was used as the dependent variable, while 25(OH)D, TC, TG, HDL, LDL, BMI, RBP4, HOMA-IR, SBP, FINS, FPG, WHR, age, sex, duration, and smoking were used as the independent variables. Multivariable logistic regression analysis showed that smoking (OR = 5.565, 95% CI = 1.379–22.458), FPG (OR = 1.818, 95% CI = 1.027–3.217), SBP (OR = 1.167, 95% CI = 1.081–1.260), and LDL (OR = 2.746, 95% CI = 1.122–6.721) were significant negative predictors. HDL (OR = 2.746, 95% CI = 0–0.432) and 25(OH)D (OR = 0.892, 95% CI = 0.813–0.978) were positive predictors. The equation is as follows:

(1) y=logitp= 0.598∗FPG+1.01∗LDL−4.25∗HDL−0.114∗25(OH)D+0.155∗SBP+1.717∗smoking−23.661.

## 4. Discussion

RBP4 is a molecule found in the circulation, thought to be secreted mainly by adipose tissue and the liver. Increased serum RBP4 levels have been reported in subjects with obesity, IR, and T2DM and in other insulin-resistant states, such as metabolic syndrome and vascular complications of DM [15]. Cabré et al. [16] pointed out that RBP4 would increase in patients of T2DM complicated with coronary heart disease, and when the level of RBP4 increased by 1/4, the risk of cardiovascular disease would increase by 2.5 times. In this study, the level of RBP4 was higher in patients of T2DM with LEAD than in patients of T2DM and healthy people. Therefore, RBP4 is involved in the pathogenesis of T2DM with LEAD. Metheniti et al. [17] reported that 25(OH)D was low in ultra obese young females and it was significantly associated with RBP4 and neutrophil gelatinase-associated lipocalin (NGAL). We found that there was a negative correlation between 25(OH)D and RBP4; the concentrations of RBP4 decreased after vitamin D supplementation in the study. Vitamin D may reduce peripheral IR by inhibiting the expression of peroxisome proliferator-activated receptor-*γ* (PPAR-*γ*) and the differentiation from preadipocytes to mature adipocytes [18]. This result suggests that one of the reasons for IR led by vitamin D deficiency in T2DM patients is the regulation of RBP4.

The third national health and nutrition examination survey (NHANES-III) found that, compared with residents with normal concentrations of 25(OH)D, residents with low concentrations of 25(OH)D had a higher incidence of peripheral arterial disease [19]. In this study, the level of 25(OH)D in T2DM group was lower than the one in control group, and it was the lowest in T2DM with LEAD group; 25(OH)D was a factor to protect DM patients from the pathogenesis of LEAD. Our findings were consistent with the research of Fahrleitner-Pammer et al. [20] about the relationship between 25(OH)D and T2DM with LEAD. The possible mechanisms of the involvement for 25(OH)D include the following. (1) Vitamin D can promote pancreatic *β* cells to secrete insulin by adjusting vitamin D receptor (VDR) and vitamin-D-dependent calcium-binding protein (DBP) in pancreatic tissue. It can also reduce IR in peripheral tissues by regulating inflammatory cytokines and inhibiting the expression of PPAR-*γ* [18]. Besides, it can affect insulin sensitivity by stimulating (PPAR*δ*) and the gene expression of insulin receptor [21]. (2) Calcitriol can regulate rennin angiotensin-aldosterone system (RAAS), inhibit the expression of renin, and adjust the synthesis and secretion of atrial natriuretic peptide. (3) Vitamin D can upregulate the expression of related protein of delaying arterial calcification including vascular endothelial growth factor and matrix metalloproteinase-9. (4) Vitamin D can reduce the occurrence of arterial calcification and atherosclerosis by inhibiting angiogenesis, smoothing muscle cells proliferation, and playing a role in immune regulation with VDR mediation of immune cells. (5) A lack of calcitriol results in an increase in the serum parathyroid hormone (PTH) levels. Excess PTH levels may at least in part promote cardiovascular disease by increasing the cardiac contractility and myocardial calcification [22]. (6) Vitamin D can improve the inflammation state [23, 24] and reduce chronic inflammatory reaction of the arterial wall [25] by inhibiting the secretion of inflammatory cytokines, such as IL-6, TNF-*α*, and C-reactive protein (CRP) [26]. (7) Calcitriol can inhibit foam cells formation in vascular wall by reducing acetylation and reduce the level of oxidized LDL in T2DM patients.

A 20-year retrospective study has found that [27] the incidence of DM complicated with cardiovascular diseases reduced by 33% in population with 800 IU vitamin D and 1200 mg calcium of daily intake compared to those with 400 IU vitamin D and 600 mg calcium of daily intake, which suggests that vitamin D supplementation might become an effective measurement to prevent the occurrences of T2DM complicated with macrovascular diseases. Major et al. [28] suggested that oral supplementation of vitamin D could regulate blood lipid. In this study, the levels of LDL and RBP4 reduced in T2DM group after the intervention of vitamin D, and, simultaneously, the incidence rate of LEAD in T2DM group also decreased after the intervention of vitamin D. Therefore, vitamin D supplementation can protect T2DM patients from being complicated with LEAD ultimately by improving IR and lipid metabolism and inhibiting inflammation.

## 5. Conclusion

There were several limitations in our study. The sample size was relatively small and we did not consider the influence of outdoor activities and seasonal variation on the level of vitamin D. However, the study was conducted in winter so that the impact of sun exposure was minimized. In conclusion, the phenomenon of vitamin D deficiency amongst T2DM patients was more and more prevalent, and, with the advantages of low price and validated long-term safety, the supplementation of vitamin D would be one of the intervening measurements for the presentation of T2DM and its vascular complications.

## Figures and Tables

**Table 1:** The comparison of basic characteristics in Groups NC, DM1, and DM2.

	NC (*n* = 38)	DM1 (*n* = 45)	DM2 (*n* = 62)
Age (y)	56.61 ± 7.45	56.24 ± 4.23	58.85 ± 6.18^b,d^
Duration (y)	—	6.45 ± 2.84	9.31 ± 4.27^d^
FPG (mmol/L)	4.65 ± 0.45	7.98 ± 1.26^b^	8.60 ± 1.24^b,c^
FINS (mU/L)	7.33 ± 1.28	8.98 ± 3.76^b^	9.22 ± 2.55^b^
TC (mmol/L)	4.78 ± 0.43	5.08 ± 0.59^a^	5.12 ± 0.43^b^
TG (mmol/L)	1.52 ± 0.42	2.52 ± 0.32^b^	2.47 ± 0.37^b^
LDL (mmol/L)	2.33 ± 0.45	2.63 ± 0.69^a^	3.10 ± 0.74^b,d^
HDL (mmol/L)	1.42 ± 0.28	1.26 ± 0.26^a^	1.11 ± 0.12^b,d^
HOMA-IR	1.52 ± 0.32	3.13 ± 1.28^b^	3.52 ± 1.02^b^
25(OH)D (ng/mL)	24.77 ± 5.92	17.32 ± 7.42^b^	12.63 ± 7.83^b,d^
RBP4 (*μ*g/mL)	14.82 ± 1.84	29.28 ± 2.23^b^	39.00 ± 2.10^b,d^
SBP (mmHg)	120 (120, 130)	134 (127, 145)^b^	153 (144, 159)^b,d^
BMI (kg/m^2^)	23.50 ± 2.07	25.61 ± 2.61^b^	24.73 ± 3.56
HbA1c (%)	5.96 ± 0.65	7.95 ± 1.21^b^	7.38 ± 0.72^b,c^
WHR	0.76 ± 0.03	0.89 ± 0.03^b^	0.87 ± 0.04^b^
Smoking (%)	9 (23.7%)	11 (24.4%)	40 (64.5%)^b,d^
Male (%)	20 (52.6%)	25 (55.6%)	38 (61.3%)

Note: compared with Group NC, ^a^ *P* < 0.05, ^b^ *P* < 0.01; compared with Group DM1, ^c^ *P* < 0.05, ^d^ *P* < 0.01.

**Table 2:** Cases with different levels of 25(OH)D in Groups NC, DM1, and DM2.

Group	25(OH)D < 20	≤20 < 25(OH)D ≤ 30	25(OH)D > 30	Chi-squared	*P*
NC	6 (15.8%)	25 (65.8%)	7 (18.4%)	38.84	<0.01
DM1	29 (64.4%)	12 (26.7%)	4 (8.9%)		
DM2	48 (77.4%)	9 (14.5%)	5 (8.1%)		

Note: NC versus DM1: *P* < 0.01; NC versus DM2: *P* < 0.01; DM1 versus DM2: *P* > 0.05.

**Table 3:** The comparison of characteristics before and after treatment in Groups DM3 and DM4.

Variable	Group DM4 (*n* = 27)			Group DM3 (*n* = 29)		
	0 w	12 w	*D*-value	0 w	12 w	*D*-value
TC (mmol/L)	5.12 ± 0.43	5.25 ± 0.32	−0.13 ± 0.47	5.17 ± 0.41	5.3 ± 0.38	−0.13 ± 0.5
TG (mmol/L)	2.47 ± 0.35	2.33 ± 0.19^b^	−0.14 ± 0.38	2.47 ± 0.40	2.50 ± 0.32	−0.03 ± 0.52
LDL (mmol/L)	3.18 ± 0.72	2.43 ± 0.41^a,b^	−0.72 ± 0.85	2.91 ± 0.73	3.03 ± 0.60	−0.12 ± 1.00
HDL (mmol/L)	1.12 ± 0.11	1.09 ± 0.26	−0.03 ± 0.27	1.11 ± 0.12	1.19 ± 0.21	−0.08 ± 0.25
25(OH)D (ng/mL)	12.39 ± 8.37	19.27 ± 2.33^a,b^	−6.89 ± 9.03^b^	13.30 ± 7.76	11.66 ± 2.37	1.63 ± 7.63
RBP4 (*μ*g/mL)	39.09 ± 2.32	25.33 ± 1.68^a,b^	13.76 ± 3.20^b^	39.02 ± 1.97	35.44 ± 2.17^a^	3.58 ± 3.05
BMI (kg/m^2^)	25.05 ± 3.30	23.95 ± 2.81	1.10 ± 4.11	24.09 ± 3.77	23.71 ± 2.85	0.38 ± 4.65
HbA1c (%)	7.38 ± 0.65	5.95 ± 0.48^a,b^	1.43 ± 0.87^b^	7.35 ± 0.78	6.74 ± 0.59^a^	0.61 ± 0.92
WHR	0.87 ± 0.04	0.87 ± 0.04	0.00 ± 0.05	0.87 ± 0.04	0.86 ± 0.03	0.01 ± 0.05

Note: comparison within the group, ^a^ *P* < 0.05; comparison between two groups, ^b^ *P* < 0.05.

**Table 4:** The analysis of the correlation between 25(OH)D and the indicators of patients in Group T2DM.

Variable	Number	*r*	*P*
Age (y)	107	−0.074	0.448
Duration (y)	107	−0.663	<0.001
FPG (mmol/L)	107	−0.229	0.018
FINS (mmol/L)	107	−0.175	0.072
TC (mmol/L)	107	−0.009	0.927
TG (mmol/L)	107	−0.082	0.403
LDL (mmol/L)	107	−0.113	0.247
HDL (mmol/L)	107	0.113	0.247
HOMA-IR	107	−0.267	0.006
RBP4 (*μ*g/mL)	107	−0.538	<0.001
SBP (mmHg)	107	−0.104	0.287
BMI (kg/m^2^)	107	−0.066	0.497
HbA1c (%)	107	−0.482	<0.001
WHR	107	−0.181	0.063

**Table 5:** Multivariable logistic regression analysis for the predictors of LEAD.

Variable	*B*	S.E.	Wals	Sig.	OR	95% CI	
						Lower	Upper
FPG (mmol/L)	0.598	0.291	4.215	0.040	1.818	1.027	3.217
LDL (mmol/L)	1.010	0.457	4.893	0.027	2.746	1.122	6.721
HDL (mmol/L)	−4.250	1.741	5.962	0.015	0.014	0.000	0.432
25(OH)D (ng/mL)	−0.114	0.047	5.933	0.015	0.892	0.813	0.978
SBP (mmHg)	0.155	0.039	15.731	0.000	1.167	1.081	1.260
Smoking	1.717	0.712	5.815	0.016	5.565	1.379	22.458
Quantity	−23.661	6.934	11.643	0.001	0.000		
